# Development of Functional Hybrid Polymers and Gel Materials for Sustainable Membrane-Based Water Treatment Technology: How to Combine Greener and Cleaner Approaches

**DOI:** 10.3390/gels9010009

**Published:** 2022-12-24

**Authors:** Giulia Rando, Silvia Sfameni, Maria Rosaria Plutino

**Affiliations:** 1 Department of Chemical, Biological, Pharmaceutical and Analytical Sciences (ChiBioFarAm), University of Messina, 98166 Messina, Italy; 2 Institute for the Study of Nanostructured Materials, ISMN—CNR, Palermo, c/o Department of ChiBioFarAm, University of Messina, 98166 Messina, Italy; 3 Department of Engineering, University of Messina, Contrada di Dio, S. Agata, 98166 Messina, Italy

**Keywords:** nano-hybrid gels, advanced materials, electrospun nanofibers, mixed-matrix membranes, water remediation, wastewater treatment

## Abstract

Water quality and disposability are among the main challenges that governments and societies will outside during the next years due to their close relationship to population growth and urbanization and their direct influence on the environment and socio-economic development. Potable water suitable for human consumption is a key resource that, unfortunately, is strongly limited by anthropogenic pollution and climate change. In this regard, new groups of compounds, referred to as emerging contaminants, represent a risk to human health and living species; they have already been identified in water bodies as a result of increased industrialization. Pesticides, cosmetics, personal care products, pharmaceuticals, organic dyes, and other man-made chemicals indispensable for modern society are among the emerging pollutants of difficult remediation by traditional methods of wastewater treatment. However, the majority of the currently used waste management and remediation techniques require significant amounts of energy and chemicals, which can themselves be sources of secondary pollution. Therefore, this review reported newly advanced, efficient, and sustainable techniques and approaches for water purification. In particular, new advancements in sustainable membrane-based filtration technologies are discussed, together with their modification through a rational safe-by-design to modulate their hydrophilicity, porosity, surface characteristics, and adsorption performances. Thus, their preparation by the use of biopolymer-based gels is described, as well as their blending with functional cross-linkers or nanofillers or by advanced and innovative approaches, such as electrospinning.

## 1. Introduction

Water is an essential asset since it is one of the most abundant resources on earth, as it covers three-quarters of its surface; about 97% of this volume is represented by seas and oceans, and only 3% is instead fresh water suitable for humans, plants, and animals. Of this fraction, almost 2.5% is contained in the polar ice caps, glaciers, and the atmosphere, leaving about 0.5–1% of the water accessible to living species in the form of rivers and groundwater [1,2,3]. This important resource is a key factor for social and economic growth, as well as for human and ecosystem health and well-being [4,5,6]. With the aim to protect, preserve, and safeguard the environment, it is increasingly necessary to move toward a sustainable lifestyle, conserving, reusing, and recycling materials at the end of their life cycle and thus reducing the waste of primary resources [7,8,9], among which is water itself. In this scenario, considerable attention has been paid to the treatment/recycling/recovery of water (underground and wastewater) for their reclamation and (re)use in order to manage a global shortage of water and the development of “water-smart” cities [10,11,12,13]. 

Unfortunately, with the growing increase in the world population and consequently in anthropological activities, water pollution is increasingly important [14,15]. Generally, a water pollutant can be defined as a physical, chemical, or biological factor that causes detrimental effects on those who consume it and on aquatic life. However, most water contaminants are found in the form of dissolved or suspended chemicals [16]. 

There are different classes of pollutants constantly released into the environment (heavy metals, oils and hydrocarbons, bacteria, etc.), but due to the increase in industrialization, new classes of substances defined as “emerging” have been detected in water bodies and represent a serious risk to human health and living species [17,18,19]. These emerging pollutants include a wide range of man-made chemicals (such as pesticides, cosmetics, personal and home care products, pharmaceuticals, organic dyes, etc.), which are in use around the world and which are indispensable for modern society [20,21,22]. It has been shown that between 1930 and 2000, the global production of man-made chemicals increased from 1 million to 400 million tons per year. According to EUROSTAT data published in 2013, between 2002 and 2011, over 50% of the total production of chemicals is represented by compounds harmful to the environment, and over 70% of these are chemicals with a significant environmental impact [23].

In this regard, wastewater treatment methods represent an important subject of investigation for the abatement not only of the common classes of pollutants, but also of various classes of emerging substances. Common wastewater treatment methods can be classified into three main categories: physical, chemical, and biological processes. In particular, a traditional wastewater treatment plant includes a combination of the three processes mentioned above to eliminate various types of pollutants [24,25]. 

Unfortunately, conventional methods of wastewater treatment and water purification are sometimes not sufficient for the removal of emerging contaminants; for this reason, new advanced techniques are increasingly studied and developed based, for example, on the use of filtration systems [26,27,28]. They are processes mainly based on the use of ceramic or polymeric-based membranes [29,30] that exploit the application of pressure for the removal of various pollutants and/or the desalination of water. 

**Figure 1:**
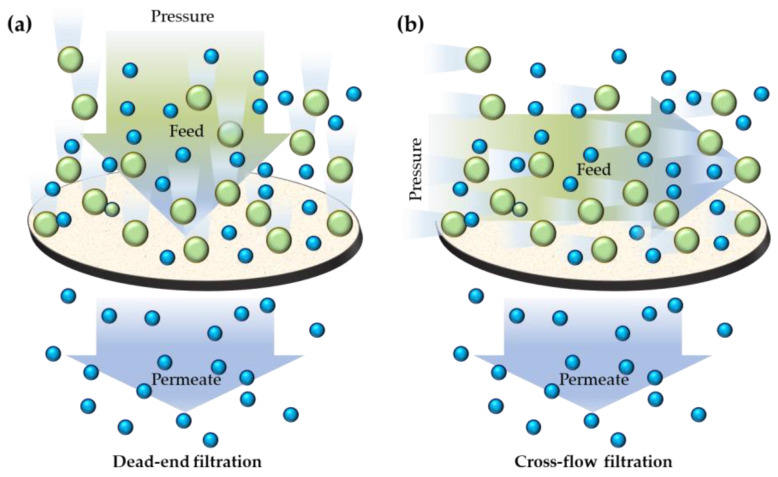
Schematization of dead-end (**a**) and cross-flow (**b**) filtration processes.

In this regard, it is possible to distinguish mainly two different approaches, as the pressure can be applied perpendicular (Figure 1a) or tangential (Figure 1b) to the membrane to perform dead-end or cross-flow filtration processes, respectively [31,32].

**Figure 2:**
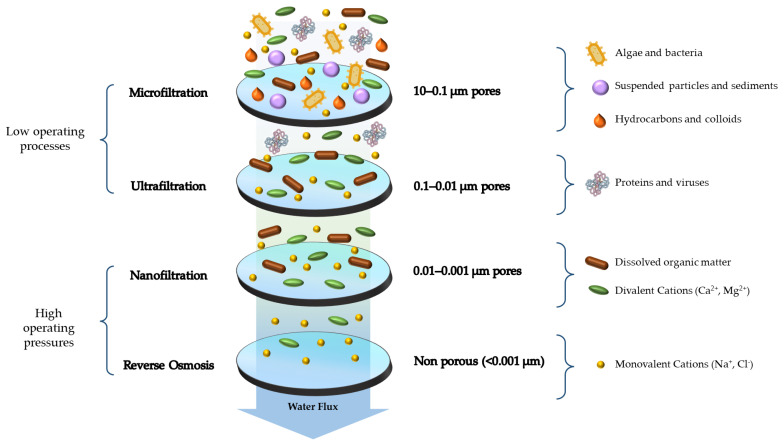
Representation of different membrane-based filtration processes with an example of the relative pollutant retention capabilities.

It is also possible to discriminate four broad categories of membranes with different contaminants-removing capacities, which is mainly related to the size of the pores of the membranes (Figure 2) [33,34,35]:

- Microfiltration membranes (with a pore size range of 0.1–5 µm), which retain species such as algae, bacteria, suspended particles, and sediments;
- Ultrafiltration membranes (with a pore size range of 0.01–0.1 µm), which retain proteins and viruses;
- Membranes for nanofiltration (with a pore size range of 0.001–0.01 µm), which retain dissolved organic substances and divalent cations;
- Reverse osmosis membranes (with a pore size range of 0.0001–0.001 µm) either have pores or are non-porous, which work on the principle of solvent diffusion across the membrane.

## 2. Overview of Membrane-Based Filtration Processes, Limitations, and Innovative/Ecofriendly Approaches

Despite some advantages and benefits of membrane technology, including the scalability, the relatively low power usage, the not necessity of chemicals such as other wastewater treatment processes, and low operating temperatures [36], the common polymers employed for the preparation of polymeric filtration membranes (listed in Table 1) came from non-renewable and petroleum resources [37,38] or toxic substances [39], representing themselves as sources of secondary pollution and hazard [36]. The main challenge of research and industries is therefore to combine greener and cleaner approaches to achieve more sustainable processes in wastewater and water treatment. 

Consequently, research is moving toward the development of new membrane filtration approaches based on the use of biopolymers or ecofriendly gel blend polymers [40,41,42,43]. Moreover, thanks to the knowledge of nanotechnology and molecular functionalization [44,45,46], it is possible to modulate the performance of these sustainable membranes that can lack in some aspects as mechanical properties and thermal and chemical resistance than the ones made from fossil-derived polymers [42,47].

**Figure 3:**
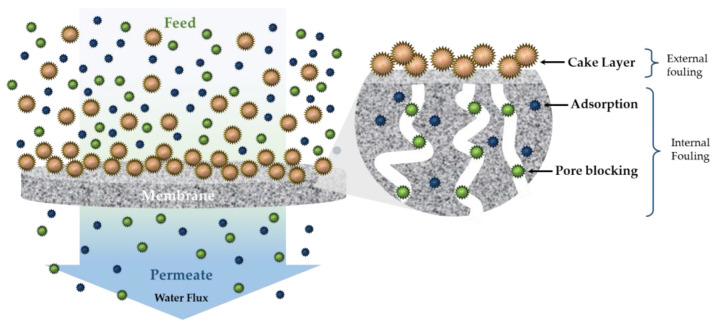
Schematization of different types of membrane fouling.

In this regard, nanohybrids and nanocomposites based on different compounds, such as metal or metal oxide nanoparticles and carbon-based or silica-based nanomaterials or sol-gel, represent a key aspect for the rational design of adsorbent materials or membranes with implemented characteristics and functional properties for water remediation and bioremediation [48]. Another aspect for which these materials came in help is the membrane fouling reduction. Fouling, which is brought on by elements such as bacteria, proteins, inorganic substances, and other organic molecules [49,50], is one of the main issues with membrane technology [51]. In particular, it can be classified as reversible and irreversible fouling and leads to the reduction of the efficiency and longevity of membranes in water and wastewater treatment processes, causing the necessity to work with higher pressures, and therefore an increment in the energy consumption and the overall process costs [52,53]. Membrane fouling can also be recognized in different forms, as shown in Figure 3 [54,55]. Nanofiltration and reverse osmosis membranes are characterized by smaller pores and are mainly affected by external fouling; meanwhile, membranes with larger pores are more interested in internal fouling [56,57]. 

**Figure 4:**
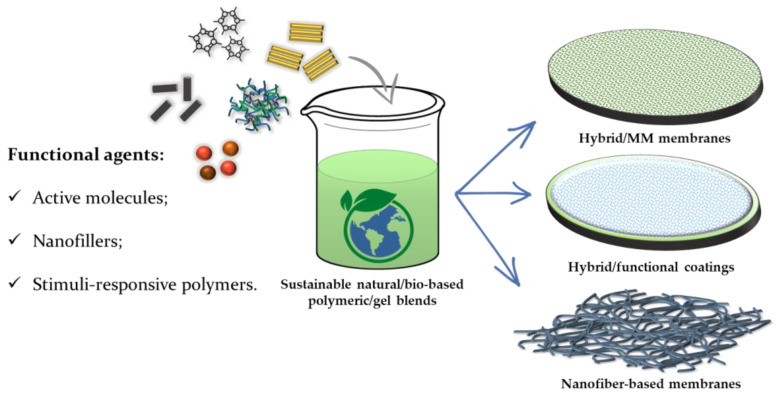
Innovative and ecofriendly approaches for water filtration membranes modification.

One of the advantages of using hybrid blend polymers stands in the opportunity to employ proper antifouling additives as membrane coating or as functional antifouling/antibacterial fillers to add to the blend gel mixture before the drying process [51]. One of the most interesting features of the gel materials is that they can be synthesized and dried at ambient temperature to obtain a final xerogel of desired porosity and shape, and either functional properties carried out, for example, by the use of an opportune functional sol-gel precursor [58]. Finally, the innovative approaches for the modification and/or preparation of natural/bio-based or ecofriendly membranes that could help to find sustainable solutions to these challenges include (Figure 4):

- The blending of bio-based polymeric/xerogel blends with different functional molecules and nanofillers to produce mixed-matrix membranes (MMM);
- The coating of commercial membranes with bio-based blends and/or xerogels doped with reinforcing or functional agents;
- The preparation of the membranes through innovative fabrication techniques such as electrospinning starting from sustainable formulations.

In the light of above-mentioned aspects, in this review a thorough overview is reported about the recent advancements on the three sustainable approaches reported in Figure 3 for the preparation and modification of ecofriendly water filtration membranes, in order to improve their performances and selectivity toward different contaminants. 

Moreover, a comparison between their filtration performances is described, in order to evaluate the most efficient approaches to achieve the future goal of reduce or replace the common non-renewable water and wastewater filtration membranes with more eco-sustainable ad functional ones obtained through a safe-by-design.

## 3. Sustainable Hybrid/Mixed-Matrix Water Filtration Membranes 

As mentioned before, one of the issues with biopolymer-based membranes can be the lack of adequate mechanical, thermal, and chemical resistance [42,47]. Therefore, their blend with other polymers, gels, functional nanofillers, or reinforcing agents is a key step. Due to their properties such as a large surface area (surface/volume ratio), size effects, reactivity, and catalytic properties, new nanotechnologies and nanomaterials are now employed in a wide range of applications, representing themselves as proper candidates to solve these problems [59,60,61,62]. In this regard, thanks to different and easy approaches, nanomaterials can be easily chemically modified, functionalized, and embedded in different polymers and matrices to obtain hybrid materials or nanocomposites with implemented chemical, physical, and mechanical properties [63,64,65,66]. Thus, they can be used to develop advanced, sustainable, stimuli-responsive, and novel products and techniques for a wide range of sectors such as blue-growth, smart and technical textiles, biomedicine, building, cultural heritages, and environmental remediation [67,68,69].

The most recent examples regarding the state of the art of water filtration membranes based on sustainable or renewable materials involve the employment of different natural-derived polymers and gels [70,71,72]. Some of the most common of them used for these purposes are listed in Table 2. 

An overview of the recent sustainable nano-hybrid and mixed-matrix membranes, based on these and other naturally derived biopolymers/-gels is discussed in this paragraph. Moreover, a comparison between their preparation methods, performances, and retention properties toward different water common and emerging pollutants, is reported in Table 3. 

Chitosan is a natural amino-polysaccharide obtained by deacetylation of chitin. It is extracted from crustaceans’ shells and after cellulose is the second most prevalent biopolymer in nature. It is characterized by a high amount of amine and hydroxyl functionalities and has drawn increasing attention in different fields as well as water treatment and purification [85,86]. In this regard, thanks to its functionalities, it represents a potential adsorbent system for different common and emerging pollutants such as heavy metals, anionic organic dyes, and macromolecules [87,88]. By easy casting or non-solvent-induced phase inversion techniques, it is possible from a gel to obtain thin films for ultra-nanofiltration approaches based on chitosan and other polymeric functional blends [40,89,90]. Additionally, due to their negatively charged surfaces, high surface area, swelling capacity (particularly the bentonite and montmorillonite clays), cation exchange capacity, and strong adsorption/absorption properties, clays find a huge application in the field of environmental remediation [91,92]. They can also be simply functionalized by varying their surface hydrophilicity/hydrophobicity and adsorption properties [93,94]. 

An example of the combination of these two materials is represented by a novel organic-inorganic hybrid thin sheet membrane obtained by a non-solvent-induced phase inversion method, starting from a chitosan/polyvinyl alcohol and montmorillonite clay blend (CS/PVA/MMT). The obtained hybrid clay-polymeric nanofiltration membrane showed suitable rejection rate of chromium and great overall performances, including increased hydrophilicity and anti-biofouling properties [79]. 

This kind of membranes, which contain evenly distributed inorganic fillers dispersed in the polymer matrix are defined as mixed-matrix membranes (MMM) [95,96]. In another study, a metal oxide nanofiller was employed for the preparation with a phase inversion method of a regenerable MMM based on cellulose acetate. In detail, the Fe–Al–Mn@chitosan nanocomposite obtained by a simple co-precipitation method from a Mn-slag waste resource, was uniformly dispersed in a cellulose acetate solution and casted by a phase inversion approach to obtain the final MMM. This last was tested in a cross-flow setup for the removal of fluoride anions. The results showed that 1 m^2^ of this MMM is capable of treating 4000 L fluoride-spiked synthetic water, exploiting adsorption and electrostatic repulsion (due to the F^−^ cake layer formed on the membrane) phenomena. Moreover, adsorption isotherm studies observed a maximum adsorption capacity of the membrane of 2.3 mg/g. Finally, the membrane was regenerated with 0.01 M NaOH to perform three cycle of filtration processes [80]. 

Beyond silica-based sol-gel nanofillers and metal oxide nanoparticles, carbon-based nanomaterials have attracted significant attention for the preparation of functional hybrid/composite materials [97,98]. A chitosan/graphene oxide (CS/GO) MMM with enhanced water permeability was obtained by a simple casting and solvent evaporation approach for the water desalination of high-salinity water by pervaporation approach [81]. Pervaporation (PV), is an ecofriendly and energy-saving approach for separating liquid mixtures. One feed component is only selectively permeated when the liquid feed comes into touch with one side of a membrane. On the other side of the membrane, the permeate, which is enhanced with this component, is removed as a vapor. The low pressure created by cooling and condensing the permeate vapor is the key factor that drives the process. Therefore, this approach came in help in an eco-sustainable and efficient way, to separate mixtures or solutions with closely spaced boiling points or azeotropes, which are challenging to separate by distillation or other methods [99,100]. 

Gel materials are widely already known adsorbent systems for different environmental pollutants [101]. In particular, microgels represent a class of useful reusable smart materials sensitive to temperature and pH stimuli, suitable for the efficient removal of heavy metal ions [102] and herbicides [103] but also for the removal and degradation of organic dyes [104]. 

Alginate is an heteropolysaccharide obtained from brown seaweed and the capsules of some bacteria that due to its tendency to form gels, is frequently used in paper, textile, food and environmental remediation sectors [105,106,107]. To improve its mechanical properties, a suitable nanofiller can be employed. In this regard, in order to increase the nanofiller dispersibility and interfacial adhesion with the substrate or polymeric matrix, also leading to the reduction of agglomeration phenomena, an organo-modification occurs. For example, through the carboxylation reaction of TiO_2_ nanoparticles, it was possible to use this organo-modified nanofiller as a proper reinforcing agent for the preparation of a hybrid hydrogel membrane based on sodium alginate (TiO_2_-COOH/CaAlg). The blend was subsequently casted by a film-casting technique and crosslinked in 2.5 wt% CaCl_2_ aqueous solution. Subsequently, the obtained negatively charged nanofiltration hydrogel membrane was employed for the rejection of different organic dyes as Brilliant blue G250, Direct black 38, and Congo red, achieving the rejections ratio of 98.4%, 96.8%, and 95.9%, respectively. This hybrid membrane also showed an increased tensile strength than the pristine sodium alginate crosslinked one and low rejection rates for different tested inorganic salts [82]. 

**Figure 5:**
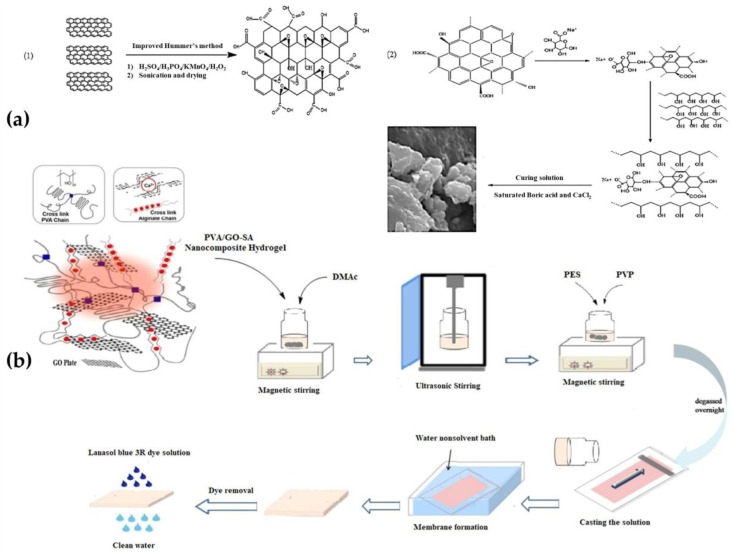
PVA-GO-NaAlg nanocomposite hydrogel production steps (**a**); description of the preparation of the functional bio-polymeric blend and final membrane (**b**). Reproduced with permission from Separation and Purification Technology; published by Elsevier, 2020 [83].

Alginate hydrogel can be also employed to blend petroleum-derived polymers in order to reduce the environmental impact of the obtained membranes, also thanks to their capability to be regenerated and reused. About that, a novel asymmetric microporous membrane based on a nanocomposite hydrogel of polyvinyl alcohol-graphene oxide-sodium alginate (PVA-GO-NaAlg) (Figure 5a) blended with PES, was produced by an immersion precipitation technique. The obtained membrane with the procedure described in Figure 5b, exhibited suitable antifouling properties and organic dyes rejection performances, in particular for the tested Lanasol Blue 3R dye [83]. There are different examples in the literature about the use of graphene oxide as an adsorbent nanofiller; it is a multilayer hydrophilic carbon nanomaterial with a very high density of charged oxygen-containing groups (i.e., alcohol, ketone, epoxide carbonyls, and carboxylic groups) that can interact with organic and inorganic pollutants in water, promoting their removal [108]. 

**Figure 6:**
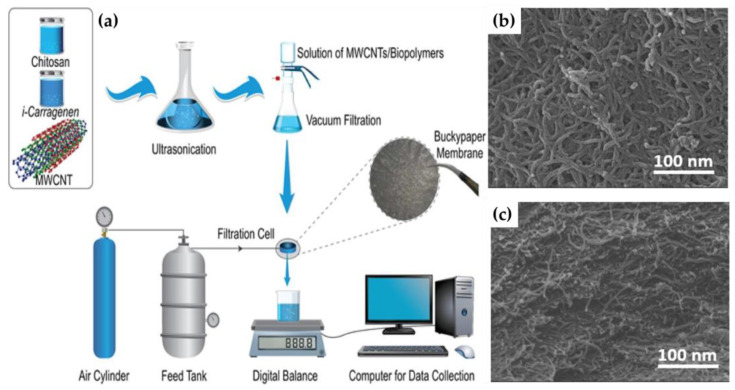
Schematic representation of the coating preparation and filtration test in the dead-end apparatus (**a**); SEM images of the MWCNTs/chitosan-carrageenan BP membrane surface (**b**) and cross-section (**c**). Reproduced with permission from Separation and Purification Technology; published by Elsevier, 2021 [84].

Among GO, other carbon-based nanomaterials as carbon nanotubes (CNTs) and multiwalled carbon nanotubes (MWCNTs), thanks to their superior properties, find as well different applications in water and wastewater treatment [109,110,111]. MWCNTs were employed to obtain a new buckypaper (BP) membrane using a vacuum filtering technique after its blending with the two biopolymers/gels chitosan and carrageenan (Figure 6a). 

Sustainable water dispersions of MWCNTs based on these two biopolymers acting as surfactants and dispersing agents for the nanofiller, were prepared and filtered through PTFE membranes. After drying the produced film was peeled off to obtain the BP membrane showing at SEM images an entangled and bundle morphology (Figure 6b,c). It was tested in a dead-end apparatus for the retention of a mixture of heavy metal ions (Cu^2+^, Cd^2+^, Co^2+^, Ni^2+^, Ba^2+^, and Pb^2+^), achieving up to 90% of their removal at low pressure and showing high permeability, remarkable selectivity, and antifouling ability [84].

Carrageenan is not only employed as surfactant for stable dispersion preparation. As a matter of fact, thanks to its ability to form stable gels, finds applications in different sectors, i.e. pharmacological, industrial, and biological [112,113]. Moreover, thanks to the sulfate, hydroxyl and carboxyl groups on its polysaccharide structure, which may serve as possible reactive and coordination sites for the adsorption of various contaminants, finds applications as adsorbent system in environmental remediation [114,115]. 

Based on the number of sulfated groups, it is possible to recognize three different forms of this biopolymer: the lambda carrageenan, which contains three of them, the iota, which contains two sulfate groups, and the kappa, which contains a single sulfate group every disaccharide unit [116].

In membrane fabrication, it can be also employed to improve the membrane hydrophilicity. For example, kappa-Cg was blended with PVDF to obtain asymmetric membrane structures with an higher dye retention and water permeability than pristine PVDF [117]. 

An ecofriendly composite membrane based on a chitosan/κ-carrageenan/acid-activated bentonite blend was obtained by a dry casting method and successfully used for the removal in a batch system of methylene blue (MB). With an adsorbent dose 0.05 g/10 mL MB solution, after 200 min at pH = 4 and temperature of 50 °C was achieved the 98% removal rate. The adsorption capacity was 18.80 mg/g at 50 °C for MB with an adsorption behavior that fits the pseudo-second-order kinetic model and Freundlich isotherm model. Furthermore, in the light of the regeneration tests, the membrane demonstrated a suitable recyclability of ∼77%, using N, N-dimethylformamide as desorbing agent [118].

Therefore, different examples of sustainable mixed-matrix, composite, and hybrid membranes were described, but despite it is possible their preparation by easy procedures, in some cases, the employed starting materials and polymers have a not treasurable economic impact in view of a large application scale. 

The blend of the commonly employed polymers with natural or more sustainable ones can represent a solution to this problem for more ecofriendly and cheaper solutions; however, in order to improve the selectivity and durability of the actual petroleum-derived membranes, it is possible to act with a more economical and effective approach, which is discussed in the next paragraph.

## 4. Hybrid/Doped Bio-Based and Functional Coatings for Filtration Membranes 

As over mentioned, a simple and sustainable approach to improve the performances of the actually employed polymeric membranes, that can lead also to and enhancement in their lifespan reducing the fouling problem, is represented by their coating with functional polymeric gels and blends [119,120]. 

Some of the most recent approaches and coating solutions are represented in Table 4, with a comparison between their preparation method and filtration performances. 

**Figure 7:**
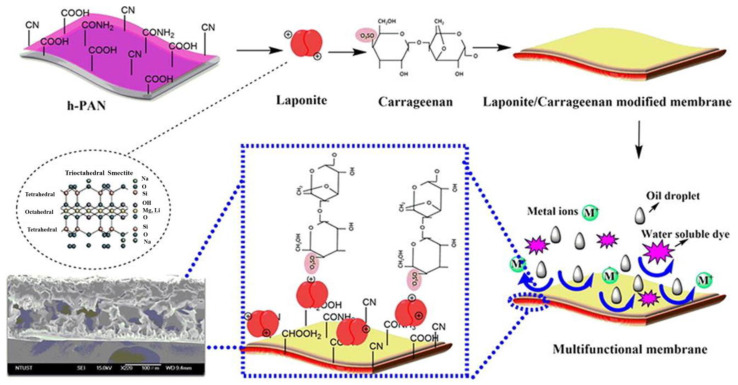
Preparation of the ĸ-carrageenan/laponite coating of h-PAN membranes with multifunctional properties. Reproduced with permission from Chemical Engineering Journal; published by Elsevier, 2020 [121].

Thanks to a rational coating design, it is in fact possible to develop functional gel coatings for the commercial membranes to confer them the capability to retain multiple pollutants [127,128,129]. For example, a hydrolyzed-PAN membrane was coated by a layer-by-layer approach with a polymeric blend prepared from naturally obtained ĸ-carrageenan and the nanoclay-laponite to achieve the preparation of an efficient self-cleaning and antifouling membrane featuring superoleophobicity properties (Figure 7). The modified laponite/ĸ-carrageenan membrane showed high water-soluble dye adsorption, in particular, of brilliant blue (BB) and rhodamine-B (RB), together with high stability and flexibility, demonstrating the efficient underwater superoleophobicity and water filtration capabilities of organic contaminants. In addition to the removal of oil emulsions and water-soluble dyes, an almost total metal ion (NaCl, MgSO_4_)-free filtrate was achieved [121]. 

**Figure 8:**
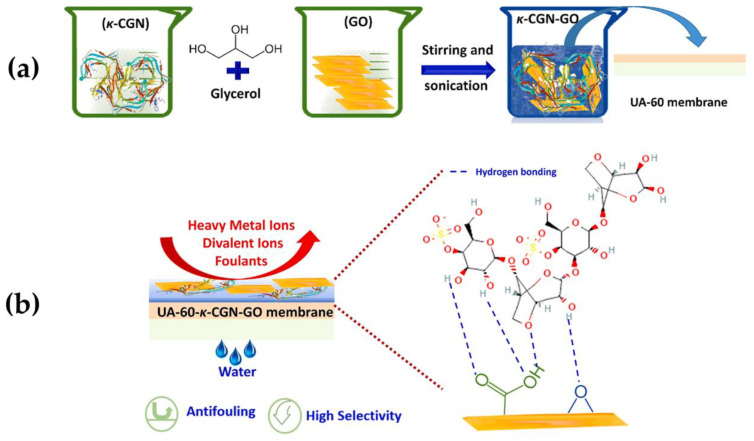
Steps for the preparation of the κ-CGN/GO composite-coated UA-60 loose nanofiltration membrane (**a**) and representation of the possible intermolecular hydrogen bonding through GO and κ-CGN with the properties and application of the final coated membrane (**b**). Reproduced with permission from Journal of Membrane Science; published by Elsevier, 2022 [125].

The negatively charged structure of k-carrageenan, leads also to improve not only the wettability, but also the salt rejection and antifouling performances of membranes. An example is represented by a crosslinked kappa-carrageenan (κ-CGN) and GO coating of a commercial UA-60 loose nanofiltration membrane. In detail, glycerol was employed as ecofriendly cross-linker for k-carrageenan. Moreover, in order to design the optimal coating, was adjusted the concentration of GO nanosheets to tailor the surface charge, hydrophilicity, and antifouling characteristics of the membrane. The coated membrane, was finally tested for the water recovery ratio and divalent ion rejection of landfill leachate wastewater (Figure 8) [125].

To achieve better performances and optimize the formulation of a functional coating, it is possible to apply a response surface methodology. On this regard, a cellulose membrane was coated by a dip-coating process with chitosan, polyethyleneimine (PEI), GO and glutaraldehyde as cross-linker. The concentration of the nanofiller and components of the bio-polymeric blend was successfully optimized through a response surface methodology to obtain a multifunctional nanocomposite coating of cellulose and glass nanofiber membrane capable of removing both positively and negatively charged heavy metals, such as Cr(VI) and Cu (II) [122].

Beyond the traditional and frequently employed activated carbon and zeolitic materials, nano metal organic frameworks (nano MOFs) represent a new class of functional molecular nanofillers that have drawn significant attention in the removal and retention of substances from water, due to their suitable water stability, large specific surface area, high porosity and the presence of unsaturated metal coordination sites [130,131,132]. A bio-polymeric blend of chitosan and aluminum fumarate (AlFu) metal organic framework, was employed as coating of a cellulose acetate forward osmosis membrane to improve the membrane hydrophilicity and therefore enhance the water flux. After pouring the polymeric blend on the membrane and the excess of solution drained, it was immersed in a crosslinking water solution of glutaraldehyde, Na_2_SO_4_ and concentrated H_2_SO_4_. This membrane was then employed for the nutrients (COD, NH_4_-N, NO_3_-N and PO_4_) concentration from synthetic and real wastewaters by forward osmosis using MgCl_2_·6 H_2_O as the draw. Reverse osmosis gets the 85% salt rejection from diluted draw solution and over 80% water recovery was achieved from forward osmosis. Moreover, were precipitated the nutrients from concentrated feed wastewater as struvite (NH_4_MgPO_4_·6 H_2_O), and by a simple physical cleaning with tap water, it was possible to clean the membrane surface from the cake layer fouling and perform more filtration cycles [123]. In another example, has been demonstrated the photocatalytic properties under the irradiation of visible light exploited from Fe_0_-doped WO_3_ nanostructures employed as nanofillers in the preparation of a functional membrane coating. In particular, thanks to a layer-by-layer approach, a commercial PES ultrafiltration membrane was coated with a chitosan sol-gel, subsequently with sodium alginate for three times, and then the Fe_0_@WO_3_ nanoparticles were deposited. The manufactured photocatalytic membranes were tested in batch and filtration systems to remove Cr(VI) ions. Under visible light illumination, the new photocatalytic membranes demonstrated considerable Cr(VI) ions elimination. This can be explained by the photocatalytic reduction of Cr(VI) ions to Cr(III) from the functional nanoparticles deposited in the external layer of the coated membrane [124].

Other natural available biopolymers/gels precursors can find different applications, in order to achieve superior membrane antifouling, hydrophilicity and chelation properties [133,134,135,136]. Their incorporation in bio-polymeric blends can involve one-step procedures to easy obtain functional gel-based coatings. A catechol/chitosan coating was simply obtained through a green approach by oxidant-induced ultrafast co-deposition on PVDF membranes. Under extreme pH environments, the obtained membrane demonstrated an excellent water permeability and strong chemical stability. Furthermore, the membrane surface hydrophilic coating featured the function of energy barrier for oil droplets, reducing the oil adhesion on the surface, leading high antifouling performances and allowing the use of the modified membrane for cyclic oil-in-water (O/W) emulsion separation processes. Additionally, a 70% higher water flux was achieved than pristine PVDF membranes and three filtration cycles were performed washing the membrane every time with DI water on a cross-flow cell [126].

Despite the chemical modulation and functionalization of membranes, innovative approaches for their preparation can include a wide range of methodologies to achieve for example micro-/nano-architecture morphologies in order to improve the membrane active surface and different other aspects [137,138]. A simple example can be represented by a PLA membrane characterized by a hierarchical surface to mimic the coral tentacle predatory behavior, for the efficient deposition of a functional nanoparticle-based coating leading to robust and superwetting performances. The process involved the micro-/nano-architecture preparation concerned the spreading and film casting of a PLA and β-cyclodextrin (β-CD) solution on a PET non-woven fabric. Subsequently, the ultrafiltration membrane obtained through a NIPS process, was dried and peeled off from the support, achieving the hierarchical surface mimicking the coral tentacles [139]. Furthermore, new and scalable methodologies, which already find an industrial application, can be applied to obtain high homogeneous systems at the nanoscale level, such as nanofibers. 

## 5. Functional/Hybrid Electrospun Nanofiber-Based Membranes 

**Figure 9:**
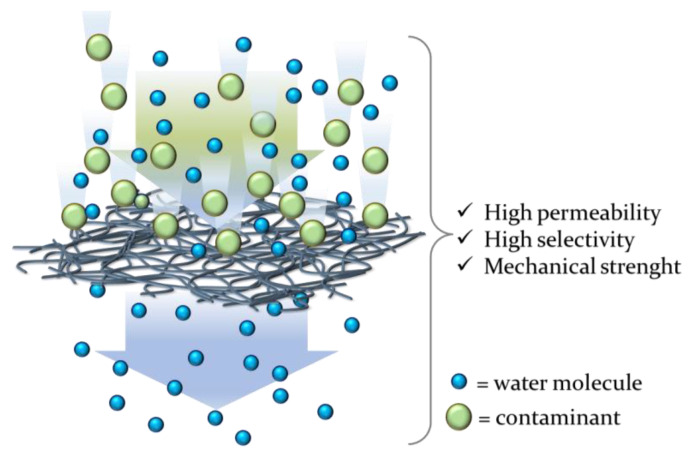
Advantages of nanofiber-based membranes for water filtration.

The use of nanofibers, i.e. fibers having a diameter less than 1 μm, is increasingly reported in the literature [140,141,142] for the preparation of water filtration membranes with implemented properties (Figure 9). Nanofibers are characterized by a high surface-to-volume ratio and have emerged as a fascinating new class of nanomaterials employed for the preparation of a wide range of materials and systems in a plenty of different sectors including energy storage, healthcare, environmental technologies, biotechnology, catalysis, air/water filtration and information technology [143,144,145,146].

**Figure 10:**
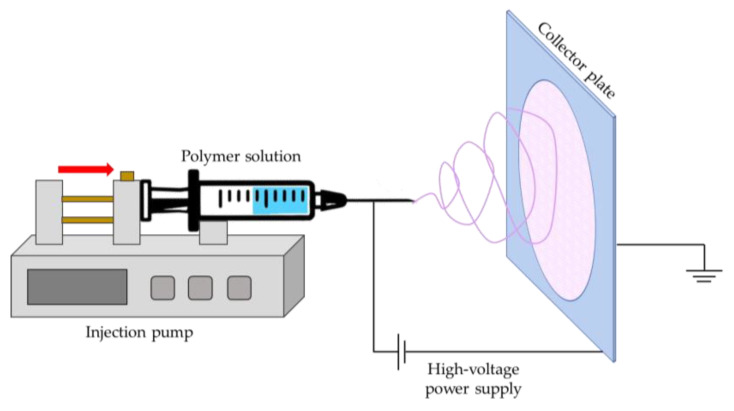
Schematization of the production of nanofibers with the electrospinning technique.

A variety of processing methods are described in the literature to produce polymeric nanofibers including drawing, self-assembly, phase separation, template synthesis and most recently electrospinning [147]. In particular, this latter is a technique that allows to easily obtain nanofibers starting from polymeric solutions or mixtures using a high voltage power supply and a collector plate, without the need of coagulating agents or high temperatures (Figure 10), for a wide range of applications [148,149,150].

To underline some of the functionalization possibilities, advantages, and versatility of this technique to achieve also functional doped sustainable nanofibers for water filtration, some examples are given in this paragraph. In Table 5, the features of recent sustainable nanofiber-based and composite membranes are summarized, together with the biopolymers, gels and functional agents employed for their preparation. 

Different examples in the literature report about the use of different supports on which electrospun the nanofibers in order to improve the efficiency and durability of the final composite membrane [157,158,159,160]. In the first work, the outer layers of a TEMPO-oxidized cellulose core-shell fiber support, were coated on both sides with electrospun nanofibers obtained from a chitosan-polyethylene oxide solution to obtain a “sandwich-like” composite membrane. It was subsequently doped with copper ions by soaking in a CuSO_4_ solution to achieve better antibacterial performances. In fact, the membrane was tested for the microfiltration of *Escherichia coli* and *Bacillus subtilis,* showing the 100% removal of both bacteria without a significant lowering of permeability and suitable reusability [151]. 

Due to its ability to transform hazardous organic contaminants into low-molecular-weight inorganic compounds, the Fenton reaction has drawn significant interest when compared to other approaches of water decontamination [161,162,163]. It is a chemical oxidation processes, that employ iron salt-based systems or most recently in heterogeneous Fenton-like methods different iron-containing materials or nano zero-valent iron, to perform catalytic degradation processes in presence of H_2_O_2_ [164,165,166]. On this regard, such materials can be combined into polymeric and gel blends to confer the functionality at the final system of perform Fenton-like degradation processes of different water pollutants. Some FeOOH/g-C_3_N_4_ submicron particles sensitive to visible light were employed to dope a PAN solution and obtain by the electrospinning process an electrospun nanofiber membrane. To improve the antifouling and hydrophilicity of the membrane it was coated with chitosan. The system was tested for the removal of methylene blue and erythromycin from water. The photo-Fenton reaction mediated from the nanofiber doped catalyst in presence of visible light and H_2_O_2_, led to great antifouling performances and contaminant removal. In particular, the degradation of the organic contaminants on the surface and pores of the membrane ensured the removal of the fouling, a stable water flux and excellent oxidation resistance leading its use for up to 10 filtration cycles [152].

MOF can be incorporated into nanofibers to be used as adsorbent systems and also for metal ions. For this purpose, a PVDF nanofibrous sublayer produced by electrospinning was used as a substrate for the electrospinning of PAN/chitosan/UiO-66-NH_2_ blends to obtain functional gel-based membranes employed for the removal of different metal ions from water. Thanks to the high surface-area-ratio of nanofibers and the properties of MOFs, the PVDF/PAN/chitosan/UiO-66-NH_2_ membrane demonstrated a high potential for the removal of metal ions from aqueous solutions, as evidenced by the high water flux and high metal ions removal within the 18 h of filtration time [153]. The approach of using nanofibrous supports for the deposition of another layer of nanofibers or a coating allows obtaining of membranes with enhanced tensile features. In this regard, they can also be coated with bio-polymeric hydrogels in order to improve the barrier, hydrophilic, and adsorption features of the composite filtration system. For example, a polyhydroxybutyrate/carbon nanotubes (PHB/CNT) electrospun nanofibrous membrane featuring high tensile mechanical properties and porosity thanks also to CNT nanofillers, was coated with sodium alginate by the simple film casting and immersion of the obtained membrane in a 1.5 wt% NaAlg solution. The membrane was subsequently immersed in CaCl_2_ aqueous solution to achieve the crosslinking of the biopolymer and form the hydrogel. In order to evaluate the adsorption and filtration performances of the composite membrane, were performed some filtration tests of brilliant blue G, direct orange S, procion red mx-5B, hydrazine yellow, and stilbene yellow dyes. These findings suggested that the composite nanofiber membrane might be used as a highly efficient nanofiltration membrane with suitable oil and protein antifouling performances for wastewater dye removal with high flux and removal rates [154]. CNTs in nanofibers not only act as reinforcing agents, but it was demonstrated how they led to the creation of nanochannels in functional coatings able to improve the water permeation and, therefore, the flux of the final membrane. This was also demonstrated from the study of some chitosan/polyvinylpyrrolidone/polyvinyl alcohol (CS/PVP/PVA) nanofiber membrane obtained through electrospinning, and then coated with the electrospray method with a CS, PVP and single-walled CNTs blend. The aligned CS/PVP/PVA electrospun membrane substrates exhibited high pure water permeate flux, a smooth surface characterized with connected pore architectures, suitable antifouling, dye rejection and heavy metals removal performances. In particular, from batch adsorption tests the maximum adsorption capacity for Cu^2+^, Ni^2+^, Cd^2+^, Pb^2+^, MG, MB, and CV were 54.32, 53.16, 52.06, 48.19, 49.31, 44.13, and 37.76 mg·g^−1^, respectively. Adsorption isotherm calculations confirmed the Langmuir model as model that fits better the results, meanwhile for Cu^2+^, Ni^2+^ the adsorption data are more in accordance with the Freundlich model [155].

**Figure 11:**
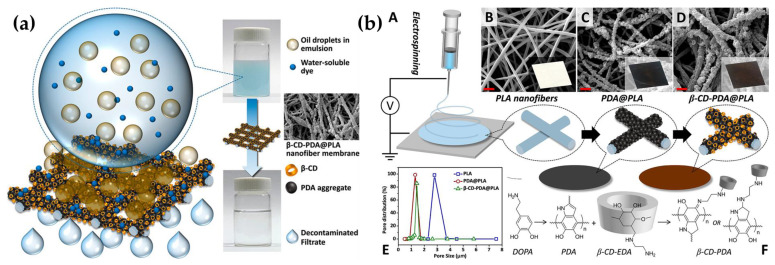
Oil-in-water separation features of the β-CD-PDA@PLA electrospun nanofiber membrane (**a**). Schematic representation of the electrospinning of the nanofiltration membrane based on β-CD-PDA@PLA (A); images of the PLA NF membrane (B), PDA@PLA NF membrane (C), β-CD-PDA@PLA NF membrane (D) taken with a camera and SEM (scale bars denote 2 µm); distribution of NF membrane pore size (E); CD-PDA@PLA NF membrane synthesis pathways (F) (**b**). Reproduced with permission from ACS Sustainable Chem. Eng.; published by ACS Publications, 2018 [156].

Therefore, were showed some recent examples of electrospun nanofiber sustainable membranes in composite systems by their preparation on supports or their use as supports for the deposition of other functional nanofibers or coatings. Despite that, it is also possible to design fully bio-based systems for multiple pollutant filtration (Figure 11a) that are not supported as the previous ones. For this purpose, some PLA nanofibers were obtained through electrospinning to be functionalized with polydopamine (PDA) and mono-6-deoxy-6-ethylenediamine-β-cyclodextrin. In particular, the PLA membrane was first functionalized with PDA and subsequently with the functional β-CDs (Figure 11b) in order to obtain the coating of the nanofibers.

The final CD-PDA@PLA nanofiltration (NF) membrane was tested for toluene-in-water emulsions separation, and methylene blue and methyl orange removal. The NF membrane exhibited superhydrophilicity and high underwater oleophobicity, with an absorbability of >95% of positively charged water-soluble organic dyes thanks to the negatively charged surface and the presence of host-guest complexation functional agents as β-CDs. Furthermore, it has excellent durability to efficiently purify the wastewater containing both toluene emulsion and MB for at least 30 cycles, as the membrane can be easily recovered by washing with a small amount of solvents and reusing for the subsequent filtration cycle [156].

Finally, the most recent examples of sustainable electrospun nanofiber membranes were described in the literature, in particular:

- Double deposited as “sandwich-like” composites;
- Deposited on commercial supports;
- Deposited on nanofibrous sublayers;
- Coated with hydrogels or functional gels;
- Coated with electrospray processes;
- Not supported.

The purpose of this paragraph was, therefore, to evaluate different recent approaches to the use of sustainable bio-based and hybrid/doped blends for the production of nanofiber membranes obtained through the electrospinning process for the removal of different pollutants from water by efficient and high flux processes, thus demonstrating the possibility of designing and easily obtaining gel-based membranes with implemented mechanical and separation features starting from bio-polymeric blends and employing a scalable approach.

## 6. Final Remarks and Conclusions

In order to answer the question if it is possible to combine greener and cleaner processes for sustainable water treatment technology, overcoming the limits of biopolymers compared to fossil-based polymers, this review explored three main sustainable approaches of biopolymer/gel-based membrane fabrication and functionalization. In Table 6 are therefore reported the advantages and advantages of the mentioned approaches for MMM adsorptive membranes based on functional polymeric and gel blends, functional polymeric/gel hybrid coatings, and electrospun nanofiber membranes.

Therefore, in light of the last years’ advancement of sustainable and green filtration approaches, as shown in this review, it is possible to underline that the rational design of the starting polymeric and gel blends with proper nanofillers or functional agents is crucial to achieving membranes with implemented mechanical, thermic and chemical resistance, but also antifouling and different pollutant retention properties. The employment of bio-based formulations and gels may lead to the possibility of carrying these sustainable approaches into large-scale applications for the filtration of industrial, municipal, ground, and in general wastewaters, thus replacing in a rational and more efficient way the conventional, more impacting membrane technologies.

## Figures and Tables

**Table 1:** Fossil-derived common polymers for usual water filtration membranes.

Polymer	Abbreviation	Chemical Structure
Polyethylene	UPE, HDPE	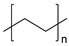
Polypropylene	PP	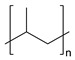
Polyvinylidene fluoride	PVDF	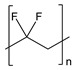
Polytetrafluoroethylene	PTFE	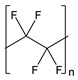
Polyacrylonitrile	PAN	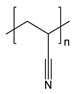
Polyethersulfone	PES	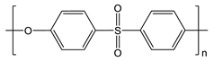
Polycarbonate	PC	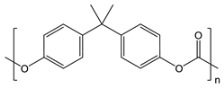
Nylon 6	Ny6	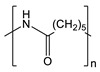
Nylon 6,6	Ny6,6	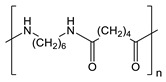

**Table 2:** Natural and renewable common polymers/gels employed for the development for water filtration membranes.

Polymer	Chemical Structure	Derivation	Ref.
Cellulose acetate ^1^	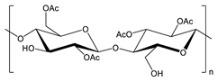	Wood pulp	[73]
Alginate ^2^	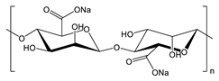	Brown algae	[74]
Chitosan ^3^	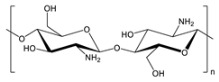	Crustacean shells	[75]
Pectin	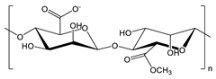	Dried citrus peels or apple pomace	[76]
Carrageenan ^4^	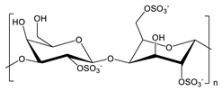	Red seaweed	[77]
Polylactic acid ^5^	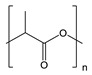	Corn starch, sugarcane, and other biomasses	[78]

^1^ Cellulose acetate = CA; ^2^ the chemical structure is referred to sodium alginate = NaAlg; ^3^ chitosan = CS; ^4^ the chemical structure is referred to lambda carrageenan = λ-Cg; ^5^ polylactic acid = PLA.

**Table 3:** Explicative table with reported a comparison of the most recent developed sustainable hybrid and polymer/gel mixed-matrix membranes.

System	Preparation Method	Filtration Process	Pollutant Treated	Filtration Performances ^1^	Ref.
CS/PVA/MMT ^2^	Non-solvent-induced phase inversion	Dead-end	Chromium	50 mg·L^−1^ feed pH = 7 100 kPa 84–88.34% removal efficiency	[79]
Fe–Al–Mn@CS CA-based	Phase inversion	Cross-flow	Fluoride anions	3.8 mg·L^−1^ feed pH = 6–9 6–8 bar Treatment capacity of 4000 L·m^−2^	[80]
CS/GO ^3^	Casting and solvent evaporation	Pervaporation	High-salinity water	5 wt% aqueous NaCl feed 81 °C 30.0 kg·m^−2^·h^−1^ permeate flux 99.99% of salt rejection	[81]
TiO_2_-COOH/CaAlg	Non-solvent-induced phase inversion	Cross-flow	Organic dyes	100 mg·L^−1^ of each dye feed 14.1 L·m^−2^·h^−1^·bar^−1^ flux 0.1 MPa Brilliant blue G250 (98.4%) and Congo Red (95.9%) removal rates	[82]
PES blended PVA-GO-NaAlg	Phase inversion by immersion precipitation	Dead-end	Organic dyes	100 mg·L^−1^ Lanasol Blue 3R pH = 4.76 3 bar Up to 88.9% dye rejection	[83]
MWCNTs/chitosan-carrageenan ^4^	Vacuum filtration	Dead-end	Heavy metals (Cu^2+^, Cd^2+^, Co^2+^, Ni^2+^, Ba^2+^, and Pb^2+^)	2 mg·L^−1^ heavy metals mixture pH = 7 1–6 bar Up to 90% removal	[84]

^1^ Are referred to optimized feed concentration, working pH and pressure, permeation flux, removal capabilities, and parameters; ^2^ MMT = montmorillonite; ^3^ GO = graphene oxide; ^4^ MWCNTs = multiwalled carbon nanotubes.

**Table 4:** Explicative table with reported a comparison of the most recent developed sustainable and functional coatings for filtration membranes.

System	Coated Membrane	Preparation Method	Filtration Process	Pollutant Treated	Filtration Performances ^1^	Ref.
ĸ-carrageenan/laponite	h-PAN	Layer-by-layer	Dead-end	Motor oil, metal ions, BB, RB ^2^	200 mg·L^−1^ BB, 100 mg·L^−1^ RB feed 100 L·m^−2^·h^−1^ flux 0.1 MPa, 27 °C >99% Hexadecane (1:30 *v*/*v*), 98% RB, 99% BB, >99% NaCl, MgSO_4_ rejection	[121]
CS, polyethyleneimine, GO	Cellulose	Dip-Coating	Batch filtration	Cr(VI) and Cu(II)	5 mL·min^−1^ feed rate 20 mL of 10 mg·L^−1^ feed ≈90% and ≈30% Cr(VI) and Cu(II) respectively	[122]
Chitosan-AlFu MOF ^3^	Cellulose acetate	Film coating	Forward osmosis cross-flow filtration	COD, NH_4_-N, NO_3_-N and PO_4_	18 L·m^−2^·h^−1^ flux for synthetic wastewater 8.75 L·m^−2^·h^−1^ flux for real wastewater Over 80% water recovery	[123]
CS-NaAlg Fe_0_@WO_3_ NPs	PES	Layer-by-layer	Cross-flow	Cr(VI)	5, 25, and 50 mg·L^−1^ feed 1 bar Irradiation chamber with visible light 99.2%, 92.1%, and 78.1% rejection, respectively	[124]
k-Cg/GO	UA-60	Film coating	Dead-end	Divalent ions	2000 mg·L^−1^ feed 5 bar 94.86% and 23.6% rejection for MgSO_4_ and NaCl, respectively	[125]
Catechol/CS	PVDF	Oxidant-induced ultrafast co-deposition	Dead-end	n-hexadecane, peanut oil, and crude oil water emulsions	0.45 g·L^−1^ of each oil and Tween 20 feed pH range 2–11 ≈428 L·m^−2^·h^−1^·bar^−1^ flux Up to 90% removal efficiencies of O/W emulsions	[126]

^1^ Are referred to optimized feed concentration, working pH and pressure, permeation flux, removal capabilities, and parameters; ^2^ BB = brilliant blue, RB = rhodamine-B; ^3^ MOF = metal organic framework.

**Table 5:** Explicative table with reported a comparison of the most recent developed sustainable and functional nanofiber-based filtration membranes.

Polymers	Doping Agent	Support	Filtration Process	Pollutant Treated	Filtration Performances ^1^	Ref.
CS/PEO ^2^	Cu^2+^	TEMPO-oxidized cellulose	Dead-end	*Escherichia coli* and *Bacillus subtilis*	104 CFU·mL^−1^ *E. coli* and *B. subtilis* feed 1600 L·m^−2^·h^−1^·MPa^−1^ flux 100% microfiltration efficiency	[151]
CS	FeOOH/g-C_3_N_4_ particles	PAN	Dead-end	MB, ERY ^3^	50 mg·L^−1^ MB, 20 mg·L^−1^ ERY feed 15 psi 35.6 L·m^−2^·h^−1^·psi^−1^ FRR 89.4% (30 min, Vis + 50 mM H_2_O_2_)	[152]
CS/PAN	UiO-66-NH_2_	PVDF nanofibrous sublayer	Cross-flow	Pb^2+^, Cd^2+^, Cr^6+^	20 mg·L^−1^ feed of metal ion 1 bar, 30 °C 452, 463, 479 L·m^−2^·h^−1^ flux, respectively 94%, 89%, 85.5% removal, respectively	[153]
CaAlg	CNTs	Polyhydroxybutyrate nanofibers	Custom filtration device	BB, DOS, PR, HY, and SY ^4^	0.1 g·L^−1^ feed of each dye 0.1 to 0.7 MPa 130 and 109.5 L·m^−2^·h^−1^ flux, BB and PR, respectively 99.1% and 97.6%, BB and PR, respectively	[154]
CS/PVP	CNTs	CS/PVP/PVA	Laboratory-scale pressure-driven membrane filtration system	Cu^2+^, Ni^2+^, Cd^2+^, Pb^2+^, MG, MB and CV ^5^	30 mg·L^−1^ feed 1 bar, 25 °C 1533.26 L·m^−2^·h^−1^ flux 95.68 %, 93.86 %, 88.52 %, 80.41%, 87.20 %, 76.33 %, 63.39 % rejection of Cu^2+^, Ni^2+^, Cd^2+^, Pb^2+^, MG, MB, CV, respectively	[155]
PLA	β-cyclodextrin	-	Dead-end	Toluene-in-water emulsions, MB, OG ^6^	Toluene-in-water emulsions of 3 wt%, 3 mg·L^−1^ MB, OG feed >1500 L·m^−2^·h^−1^ flux >95% oil/water separation efficiency	[156]

^1^ Are referred to optimized feed concentration, working pH and pressure, permeation flux, removal capabilities, and parameters; ^2^ PEO = polyethylene oxide; ^3^ MB = methylene blue, ERY = erythromycin; ^4^ DOS = direct orange S, PR = procion red mx-5B, HY = hydrazine yellow, SY = stilbene yellow, ^5^ MG = malachite green, CV = crystal violet; ^6^ OG = methyl orange.

**Table 6:** Comparison of the advantages and disadvantages of the technologies and approaches related to sustainable filtration membranes described in this review.

System	Advantages	Disadvantages
Mixed-matrix membranes	High filtering performances Long-lasting operations Capability to absorb different types of impurities from water Superior antimicrobial qualities Easy reusability Easy management	Development by starting materials and polymers with a not treasurable economic impact in view of large application scales
Functional coated membranes	Improved filtering performance of commercial membranes Implemented hydrophilicity Antifouling features Longer usage life of commercial membranes Lower operational pressures	Homogeneity of the coating related to the deposition method Operational pressures still limited by the commercial membrane employed
Electrospun nanofiber membranes	Obtainement thorough easy electrospinning processes High tensile strength High operational flux High porosity Modulable functionalities Modulable dimension of the nanofibers Easy post-functionalization with simple immersion in functional gel blends or by electrospray processes	Need of optimized and suitable protocols of electrospinning to achieve high stable processes for large-scale production

## Data Availability

Not applicable.
